# Dyslipidemia versus obesity as predictors of ischemic stroke prognosis: a multi-center study in China

**DOI:** 10.1186/s12944-024-02061-9

**Published:** 2024-03-09

**Authors:** Hang Ruan, Xiao Ran, Shu-sheng Li, Qin Zhang

**Affiliations:** 1 Department of Critical-care Medicine, Tongji Hospital, Tongji Medical College, Huazhong University of Science and Technology, Wuhan, 430030 China; 2 Department of Emergency Medicine, Tongji Hospital, Tongji Medical College, Huazhong University of Science and Technology, Wuhan, China; 3 Department of Anesthesiology, Hubei Key Laboratory of Geriatric Anesthesia and Perioperative Brain Health, and Wuhan Clinical Research Center for Geriatric Anesthesia, Tongji Hospital, Tongji Medical College, Huazhong University of Science and Technology, Wuhan, 430030 China

**Keywords:** Obesity, Stroke, Triglyceride, Total cholesterol, Machine learning

## Abstract

**Background:**

This multicenter observational study aimed to determine whether dyslipidemia or obesity contributes more significantly to unfavorable clinical outcomes in patients experiencing a first-ever ischemic stroke (IS).

**Methods:**

The study employed a machine learning predictive model to investigate associations among body mass index (BMI), body fat percentage (BFP), high-density lipoprotein (HDL), triglycerides (TG), and total cholesterol (TC) with adverse outcomes in IS patients. Extensive real-world clinical data was utilized, and risk factors significantly linked to adverse outcomes were identified through multivariate analysis, propensity score matching (PSM), and regression discontinuity design (RDD) techniques. Furthermore, these findings were validated via a nationwide multicenter prospective cohort study.

**Results:**

In the derived cohort, a total of 45,162 patients diagnosed with IS were assessed, with 522 experiencing adverse outcomes. A multifactorial analysis incorporating PSM and RDD methods identified TG (adjusted odds ratio (OR) = 1.110; 95% confidence interval (CI): 1.041–1.183; *P* <  0.01) and TC (adjusted OR = 1.139; 95%CI: 1.039–1.248; *P* <  0.01) as risk factors. However, BMI, BFP, and HDL showed no significant effect. In the validation cohort, 1410 controls and 941 patients were enrolled, confirming that lipid levels are more strongly correlated with the prognosis of IS patients compared to obesity (TC, OR = 1.369; 95%CI: 1.069–1.754; *P* <  0.05; TG, OR = 1.332; 95%CI: 1.097–1.618; *P* <  0.01).

**Conclusion:**

This study suggests that dyslipidemia has a more substantial impact on the prognosis of IS patients compared to obesity. This highlights the importance of prioritizing dyslipidemia management in the treatment and prevention of adverse outcomes in IS patients.

**Graphical abstract:**

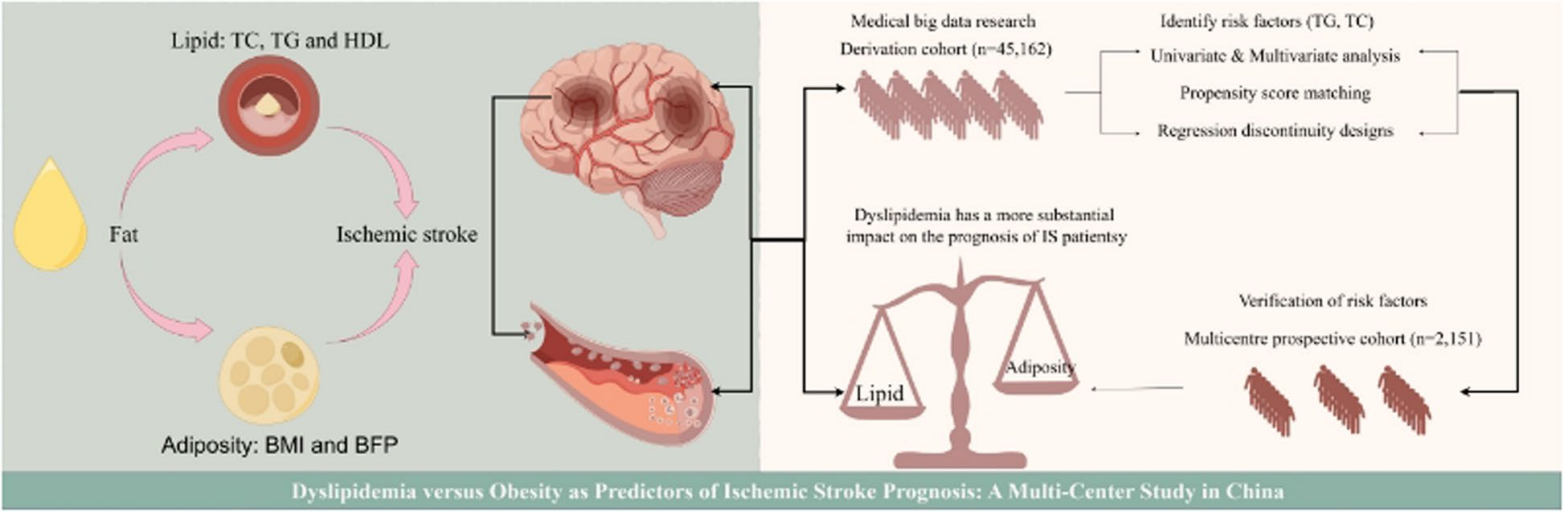

**Supplementary Information:**

The online version contains supplementary material available at 10.1186/s12944-024-02061-9.

## Background

Ischemic stroke (IS) remains a significant challenge in intensive care and ranks as the second leading cause of global mortality and morbidity [1, 2]. Historically, obesity has been linked to an unfavourable prognosis in patients with ischemic stroke, encompassing outcomes such as stroke-related mortality and recurrence. However, recent studies have highlighted a paradoxical observation of improved prognosis in obese patients compared to underweight and normal-weight individuals, known as the “obesity paradox” [3, 4]. This phenomenon suggests that obesity may not be unequivocally associated with negative health outcomes in the ischemic stroke population. Additionally, individuals with obesity may exhibit localized fat accumulation, such as excess subcutaneous and visceral fat, as well as elevated blood lipid levels, including various dyslipidaemias. These factors may be present in patients individually or concurrently, prompting the need to discern the relationship between prognosis in IS patients and obesity, and abnormal blood lipid levels.

Current studies have endeavoured to untangle the individual associations of lipid levels and obesity with IS risk, yet these clinical investigations have yielded conflicting results [5–7]. For instance, Ovbiagele et al. [6] conducted a 2.5-year follow-up study involving 20,332 patients with recent IS and found that obesity was not significantly related to the risk of recurrent stroke, contrasting a cohort study by Andersen et al. [7], which observed a significantly lower risk of readmission for recurrent stroke in obese patients. Given the rising prevalence of ischemic stroke (IS) and the unique genetic and lifestyle attributes of its demographic, it is essential to investigate the individual contributions of lipid levels and body mass index in forecasting prognosis following an ischemic stroke [8, 9].

Traditionally, obesity has been primarily measured using the body mass index (BMI), a recognized indicator of obesity that has also been linked to the development of cardiovascular risk factors and stroke incidence [10, 11]. From the BMI metric, the body fat percentage (BFP) is derived and employed in calculating a patient’s body fat percentage [12]. For lipid assessment, total cholesterol (TC) and triglycerides (TG) act as the primary risk indicators. Lipid levels, including cholesterol and triglycerides, have long been associated with atherosclerosis and ischemic stroke pathogenesis [13–15]. This study aims to compare the indicators and gather additional covariates to assess whether obesity or elevated lipids are the predominant factors contributing to poor prognosis in IS patients.

This study proposed the hypothesis that dyslipidaemia, rather than obesity, may be a significant risk factor for adverse prognosis in individuals who have suffered a stroke. Data from three medical centers overseeing 45,162 stroke patients were collated to scrutinize five obesity indices – specifically BMI, BFP, TC, TG, and High-Density Lipoprotein (HDL), and their preliminary associations with poor prognosis. Furthermore, propensity score matching (PSM) and regression discontinuity design (RDD) were employed to ascertain the connections between different fat indicators and poor prognosis. Finally, the associations between fat indicators and adverse outcomes were further substantiated by corroborating the results with data from a previously conducted national multicentre prospective cohort study [16].

## Methods

### Study designs

**Fig. 1:**
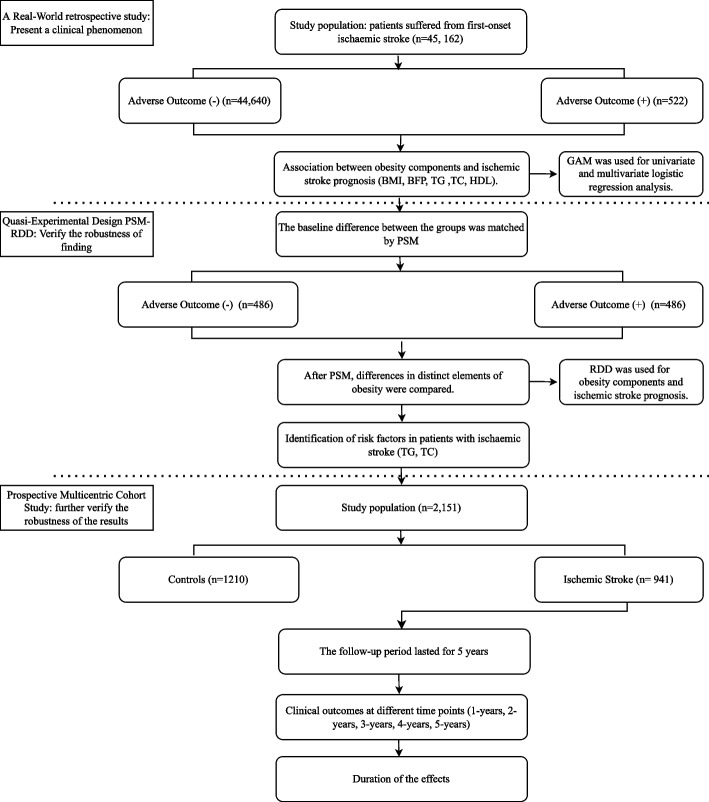
Flowchart of study design

The study involved two separates’ cohorts: the derivation cohort and the validation cohort. The primary objective was to utilize the derivation cohort to initially investigate the correlation between various fat metrics and adverse outcomes. Subsequently, the aim was to validate these findings in a separate validation cohort and evaluate the influence of fat metrics on clinical outcomes at various time intervals. The details of the study’s design are depicted in Fig. 1.

### Study population and study endpoints

The study population included patients who had experienced their first-ever IS, diagnosed according to the International Classification of Diseases, Ninth Revision [17]. All subjects were evaluated using rigorous neuroimaging techniques, such as head computed tomography and/or magnetic resonance imaging, and the images were reviewed by two or more experienced neuroradiologists. Adverse outcomes were defined as composite endpoint events, including mortality and recurrences. The primary endpoint was adverse outcomes, and the secondary endpoint was recurrence-free survival (RFS).

### Inclusion/exclusion criteria and study variables for the derivation cohort

Clinical data for the derivation cohort were obtained from three branch areas of the Tongji Hospital. These data were extracted from Tongji Hospital’s electronic medical records, the largest healthcare facility in the Hubei region, China. Their extensive system contains over 60 million medical records dating back to 1980. Information on patients admitted for their first-ever ischemic stroke from January 2012 to January 2022 was collected retrospectively. The study excluded individuals under 18 years of age and pregnant women. The variables collected included the primary test parameters of patients, covering age, gender, smoking and alcohol consumption status, arterial blood pressure, fat-related indicators, and comorbidities.

Obesity, traditionally defined as abnormal accumulation of adipose tissue with potential health consequences [18, 19], has often been characterized using BMI in clinical studies [1, 6, 7, 20–24]. However, BMI alone is insufficient due to its inability to differentiate fat from muscle mass and consider factors like age, gender, and ethnicity [12, 25]. This study expanded fat-related indicators to include BFP, HDL, TG, and TC, offering a more comprehensive evaluation of the impact of fat on patients.

The BMI categories were determined according to Chinese standards as follows: underweight: BMI < 18.5 kg/m^2^; normal range: 18.5 ≤ BMI < 24 kg/m^2^; overweight: BMI ≥ 24 kg/m^2^. The fat metrics considered for this study comprised BMI, BFP, HDL, TG, and TC. The BFP is determined using the Formula 1 [12, 26].

$$\textrm{Formula}\ 1:\textrm{BFP}=\left(\left({1.20}^{\ast }\ \textrm{BMI}\right)+\left({0.23}^{\ast }\ \textrm{Age}\right)\hbox{-} {10.8}^{\ast }\ \textrm{Sex}\hbox{-} 5.40\right)\ \left(\textrm{Sex}:\textrm{male}=1,\textrm{female}=0\right)$$

### Validation cohort

A national multicentre prospective cohort study from 5 centres in China was used as the validation cohort. This validation group consisted of 1210 controls and 941 patients with an initial IS, monitored for 5 years. The study excluded patients with concomitant chronic kidney disease or chronic respiratory disease. Further details are disclosed in previous studies [16].

### Clinical correlation analysis

The correlation between fat-related markers and adverse outcomes in IS was investigated using local weighted regression scatterplot smoothing (LOWESS) and trend tests. Nonlinear correlations were identified through restricted cubic spline regression (RCS). The connections between covariates and outcomes were assessed utilizing a generalized additive model (GAM) fitted by maximizing the penalized likelihood with a binomial family and logit link functions [27]. In the univariate analysis, each potential risk factor was individually assessed for its association with the outcome variable. Factors demonstrating a statistically significant association (*P* <  0.1) were further evaluated in the multivariate analysis.

### Machine learning and quasi experimental design

Five machine learning models—GaussianNB (GNB), LogisticRegression (LR), DecisionTreeClassifier (DTC), RandomForestClassifier (RF), and GradientBoostingClassifier (GBC)—were employed to develop a predictive model for forecasting adverse outcomes in IS based on fat-related metrics. The assessment was based on the area under the receiver operating characteristic (ROC) curve and model performance to determine the most effective risk prediction model. Subsequently, the SHapley Additive exPlanations (SHAP) value theory was leveraged to delineate the impact of different obesity indicators on adverse outcomes in IS patients [28].

Associations between obesity indicators and prognosis were examined using univariate and multivariate logistic regression, as well as PSM and RDD [20, 29]. RDD is a quasi-experimental design providing insights into the practical effects of treatments and strategies in real-world settings [20, 30–32]. Covariate selection and combination for PSM were performed using the “psestimate” command [33]. The primary objective of the program was to select a linear or quadratic function of covariates for inclusion in the estimation function of the propensity score.

### Statistical analysis

The analysis utilized R software (version 4.3.0), Stata 17.0 software (StataCorp LP, College Station, TX), and employed machine learning through Anaconda3 software. For numerical variables with a sample size ≤5000, either the mean ± standard deviation or the median (upper and lower quartiles) were computed, selected based on the data distribution’s normality. Meanwhile, the mean ± standard deviation was calculated for numerical variables exceeding a sample size of 5000. Categorical variables were presented as frequencies and percentages. Comparison of continuous variables between two groups was executed using t-tests for normally distributed data with homogeneity of variance, and for three-group comparisons under similar conditions, one-way analysis of variance (ANOVA) was employed. In cases where normal distribution was met, but not the homogeneity of variance test, comparisons were made using the Welch t-test and Welch one-way ANOVA for two- and three-group analyses, respectively. For non-normally distributed data, two- and three-group comparisons were performed using the Wilcoxon test and Kruskal-Wallis test, respectively. The chi-square test was applied to assess the comparison of categorical variables between groups, with statistical significance set at two-tailed *P* <  0.05. Furthermore, the odds ratio (OR) and the associated 95% confidence interval (CI) values were calculated. Additionally, the Kaplan-Meier (KM) curves were used to analyse survival data, providing visualization and comparison of the survival experience across different groups over time.

### Ethical considerations

This study received approval from the Institutional Review Board of the Tongji Hospital, Tongji Medical College, Huazhong University of Science and Technology (Wuhan, China; Approval No. TJ-I TJ-IRB20230830) and adhered to the principles of the Declaration of Helsinki. Written informed consent was waived due to the observational nature of the study.

## Results

### Demographic and clinical characteristics of the study population

The study population’s demographic and clinical characteristics are presented in Table 1, representing the derivation cohort of 45,162 individuals diagnosed with IS, with an average age of 60.4 ± 14.4 years. Among them, males constituted the majority at 61.31%, with 30.72% being smokers and 20.03% alcohol consumers. Adverse outcomes were observed in 1.16% of this cohort. Patients were categorized into two groups based on the occurrence of adverse outcomes during hospitalization. Significant differences in HDL, BFP, TC, and TG were observed between the two groups concerning obesity indicators (all *P* <  0.01), while no significant disparity was found in BMI (*P* > 0.05). Baseline characteristics of the validation cohort are presented in Supplementary Table 1.

**Table 1:** Baseline patient characteristics of individuals with ischemic stroke in the derivation cohort

Characteristics	Total (*n* = 45,162)	Adverse Outcome (−) (*n* = 44,640)	Adverse Outcome (+) (*n* = 522)	*P*-value
Patient status				
Age, years	60.4 ± 14.4	60.3 ± 14.3	72.1 ± 14.8	< 0.001
Sex male, n (%)	27,690 (61.31%)	27,329 (61.22%)	361 (69.16%)	< 0.001
Smoker, n (%)	13,873 (30.72%)	13,692 (30.67%)	181 (34.67%)	0.049
Drinker, n (%)	9046 (20.03%)	8926 (20.00%)	120 (22.99%)	0.089
SBP, mmHg	133.5 ± 21.8	133.5 ± 21.7	126.1 ± 24.0	< 0.001
DBP, mmHg	81.0 ± 13.0	81.1 ± 13.0	73.4 ± 15.2	< 0.001
Fat indicators				
TC, mmol/L	4.41 ± 1.09	4.41 ± 1.08	4.77 ± 1.26	< 0.001
TG, mmol/L	1.76 ± 1.25	1.75 ± 1.24	2.11 ± 1.61	< 0.001
HDL, mmol/L	1.12 ± 0.30	1.12 ± 0.30	1.18 ± 0.37	< 0.001
BFP, %	30.7 ± 5.8	30.6 ± 5.8	32.5 ± 5.8	< 0.001
BMI, kg/m^2^				0.291
< 18.5	580 (1.28%)	572 (1.28%)	8 (1.53%)	
18.5–24	20,579 (45.57%)	20,325 (45.53%)	254 (48.66%)	
≥ 24	24,003 (53.15%)	23,743 (53.19%)	260 (49.81%)	
Comorbidities				
Diabetes, n (%)	10,885 (24.10%)	10,705 (23.98%)	180 (34.48%)	< 0.001
Hypertension, n (%)	24,496 (54.24%)	24,146 (54.09%)	350 (67.05%)	< 0.001
CHD, n (%)	13,173 (29.17%)	12,827 (28.73%)	346 (66.28%)	< 0.001
CKD, n (%)	4477 (9.91%)	4306 (9.65%)	171 (32.76%)	< 0.001
CRD, n (%)	18,171 (40.24%)	17,754 (39.77%)	417 (79.89%)	< 0.001
Treatment				
Aspirin use, n (%)	17,130 (40.15%)	16,940 (37.95%)	190 (36.40%)	0.380
Clopidogrel use, n (%)	13,259 (31.08%)	13,095 (29.33%)	164 (31.42%)	0.352
Thrombolytic, n (%)	462 (1.08%)	446 (1.00%)	16 (3.07%)	< 0.001
Insulin use, n (%)	7080 (16.59%)	6827 (15.29%)	253 (48.47%)	< 0.001
Antihypertensive use, n (%)	17,410 (38.55%)	17,190 (38.51%)	220 (42.15%)	0.090
Statin use, n (%)	20,278 (47.53%)	20,025 (44.86%)	253 (48.47%)	0.129
Oxygen therapy, n (%)	12,799 (28.34%)	12,563 (28.14%)	236 (45.21%)	< 0.001

*BFP* Body fat percentage, *BMI* Body Mass Index, *CHD* Chronic cardiac disease, *CKD* Chronic Kidney Disease, *CRD* Chronic respiratory disease, *DBP* diastolic blood pressure, *HDL* High-Density Lipoprotein, *SBP* Systolic blood pressure, *TC* Total Cholesterol, *TG* Triglyceride

The demographic and clinical data were presented as column percentages (%)

### Non-linear association of fat-related indicators with adverse outcomes

**Fig. 2:**
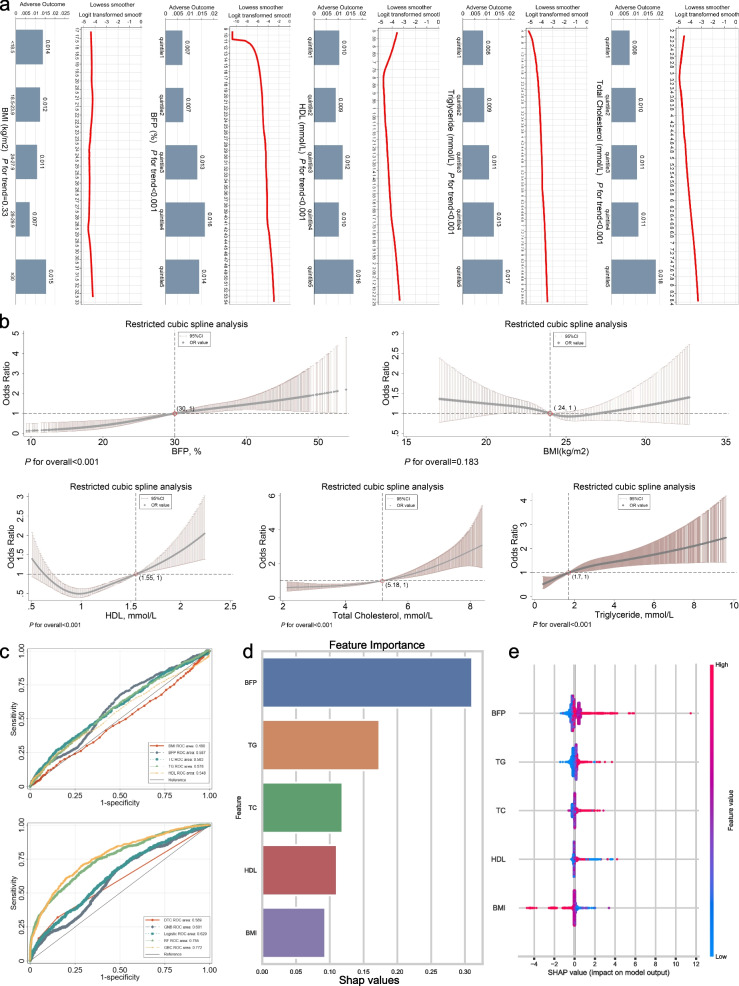
Exploratory analysis of the correlation between fat-related indicators and clinical outcomes. **a** The LOWESS curves depict the associations between fat-related indicators and clinical outcomes, while the box diagram reveals variations in clinical outcomes observed across quintiles of the indicators. **b** Curves from the restricted cubic spline regression depicting various fat-related indicators and their impact on clinical outcomes. **c** ROC curves for fat-related metrics in predicting clinical outcomes and ROC curves for machine learning-based methods in prognosticating clinical outcomes. **d** Ranking the importance of various obesity indicators in Gradient Boosting Classifier machine learning models. **e** Summary plot of SHAP values for the predictive features in the Gradient Boosting Classifier model. In the SHAP summary plot, each row represents a feature, and each point represents a sample. The color denotes the magnitude of the feature value, with red indicating high values and blue indicating low values. Positive SHAP values indicate a positive impact of the feature on the model, while negative values indicate a negative impact. Abbreviations: SHAP, Shapley’s additive interpretation

As illustrated in Fig. 2a, the LOWESS regression suggests a non-linear relationship between fat-related indicators, excluding BMI, potentially demonstrating a non-linear association with adverse outcomes (all *P* for trend< 0.001). The RCS regression further validated the non-linear relationship between fat-related indicators, excluding BMI, and adverse outcomes (*P* for overall< 0.001, Fig. 2b). The ROC curves revealed a positive correlation between the fat indicators and adverse outcomes (Area Under Curve (AUC) > 0.5, Fig. 2c), except for BMI, which exhibited a negative correlation with adverse outcomes (AUC = 0.480, Supplementary Table 2).

Supplementary Fig. 1 shows the process of parameter tuning for machine learning. The GBC model achieved optimal predictive performance in the machine learning model based on fat-related clinical indicators (AUC = 0.772, 95%CI (0.751–0.793), Supplementary Table 3). The SHAP suggested that BFP made the highest contribution to adverse outcomes, while BMI contributed the least (Fig. 2d). Higher BMI and HDL were found to be protective against adverse outcomes, while elevated levels of triglycerides, total cholesterol, and BFP were identified as risk factors for adverse outcomes in IS patients (Fig. 2e).

### TG and TC was an independent risk factor against adverse outcome

In univariate analyses, all fat-related indicators, except BMI, were identified as risk factors (all OR > 1, and *P* <  0.001, Table 2). The subsequent multivariate analysis utilized GAM regression, enabling the simultaneous assessment of multiple potential risk factors while adjusting for confounding variables. The multivariate analysis revealed that both TG (adjusted OR = 1.110, 95% CI: (1.041–1.183), ***P*** = 0.001) and TC (adjusted OR = 1.139, 95% CI: (1.039–1.248), ***P*** = 0.005) were significantly associated with adverse outcomes as independent risk factors. Additionally, no multicollinearity was observed (Supplementary Table 4).

**Table 2:** Univariate & Multivariate analyses for the adverse outcome in the derivation cohort

Characteristics	OR (95% CI) Univariate analysis	*P*-value	OR (95% CI) Multivariate analysis	*P*-value
Age, years	1.075 (1.066–1.082)	<0.001	1.064 (1.051–1.077)	< 0.001
Sex, Female vs Male	1.420 (1.178–1.711)	<0.001	1.097 (0.686–1.756)	0.699
Smoker, No vs Yes	1.200 (1.001–1.438)	0.049	0.831 (0.653–1.058)	0.133
Drinker, No vs Yes	1.194 (0.973–1.466)	0.090	0.893 (0.690–1.158)	0.394
SBP, mmHg	0.983 (0.979–0.987)	<0.001	0.992 (0.986–0.998)	0.011
DBP, mmHg	0.952 (0.945–0.959)	<0.001	0.988 (0.978–0.998)	0.016
BFP, %	1.052 (1.037–1.068)	<0.001	0.981 (0.942–1.021)	0.339
BMI, kg/m^2^				
18.5–24	Ref.	Ref.		
<18.5	1.119 (0.551–2.273)	0.756		
≥24	0.876 (0.736–1.043)	0.137		
TC, mmol/L	1.322 (1.230–1.421)	<0.001	1.139 (1.039–1.248)	0.005
TG, mmol/L	1.180 (1.123–1.241)	<0.001	1.110 (1.041–1.183)	0.001
HDL, mmol/L	1.935 (1.482–2.527)	<0.001	1.345 (0.978–1.850)	0.068
Diabetes, n (%)	1.668 (1.391–2.001)	<0.001	0.684 (0.551–0.851)	0.001
Hypertension, n (%)	1.727 (1.438–2.075)	<0.001	0.974 (0.783–1.211)	0.813
CHD, n (%)	4.876 (4.062–5.852)	<0.001	1.914 (1.555–2.356)	<0.001
CKD, n (%)	4.563 (3.791–5.493)	<0.001	1.532 (1.236–1.899)	<0.001
CRD, n (%)	6.014 (4.851–7.455)	<0.001	2.712 (2.140–3.438)	<0.001
Aspirin use, No vs Yes	0.922 (0.768–1.106)	0.380		
Clopidogrel, No vs Yes	1.093 (0.906–1.320)	0.352		
Thrombolytic, No vs Yes	3.112 (1.875–5.165)	<0.001	1.091 (0.636–1.872)	0.753
Insulin use, No vs Yes	5.368 (4.494–6.412)	<0.001	3.338 (2.709–4.113)	<0.001
Statin use, No vs Yes	1.147 (0.961–1.368)	0.130		
Antihypertensive use, No vs Yes	1.163 (0.977–1.385)	0.090		
Oxygen therapy, No vs Yes	2.107 (1.771–2.506)	<0.001	1.156 (0.956–1.399)	0.136

Following PSM (Supplementary Fig. 2), it was observed that the levels of TG (1.71 (1.17, 2.4) vs. 1.47 (1.02, 2.13), *P* <  0.001) and TC (4.60 (3.93, 5.59) vs. 4.51 (3.82, 5.30), *P* = 0.033) were significantly increased in patients experiencing adverse outcomes (Table 3).

**Table 3:** Baseline patient characteristics of individuals with ischemic stroke after PSM

Characteristics	Adverse Outcome (+) (*n* = 486)	Adverse Outcome (−) (n = 486)	*P*-value
Patient status			
Age, years	74 (61, 83)	75 (65, 83)	0.384
Sex male, n (%)	331 (68.11%)	339 (69.75%)	0.579
Smoker, n (%)	173 (35.60%)	177 (36.42%)	0.789
Drinker, n (%)	114 (23.46%)	109 (22.43%)	0.703
SBP, mmHg	123 (110, 139)	123.5 (109, 139.75)	0.761
DBP, mmHg	71 (63, 84)	71 (63, 81)	0.501
Comorbidities			
Diabetes, n (%)	171 (35.19%)	176 (36.21%)	0.738
Hypertension, n (%)	327 (67.28%)	336 (69.14%)	0.535
CHD, n (%)	318 (65.43%)	304 (62.55%)	0.350
CKD, n (%)	154 (31.69%)	158 (32.51%)	0.783
CRD, n (%)	386 (79.42%)	393 (80.86%)	0.574
Treatment			
Thrombolytic, n (%)	15 (3.09%)	8 (1.65%)	0.140
Insulin use, n (%)	243 (50.00%)	225 (46.30%)	0.248
Oxygen therapy, n (%)	224 (46.09%)	219 (45.06%)	0.747
Fat-related indicators			
BMI, kg/m^2^	23.9 (23, 24.7)	24 (23.2, 24.7)	0.693
BFP, %	31.7 (28.5, 36.3)	31.6 (28.7, 35.5)	0.996
TC, mmol/L	4.60 (3.93, 5.59)	4.51 (3.82, 5.30)	0.033
TG, mmol/L	1.71 (1.17, 2.4)	1.47 (1.02, 2.13)	<0.001
HDL, mmol/L	1.12 (0.95, 1.39)	1.12 (0.95, 1.32)	0.569

**Fig. 3:**
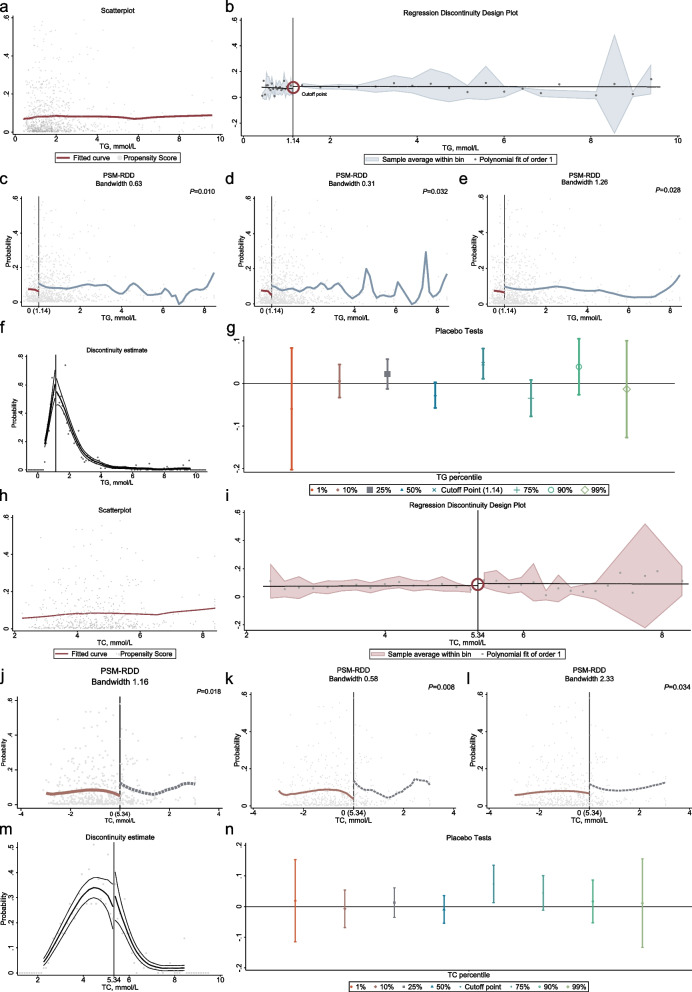
Analyzing the Relationship Between TC, TG, and Adverse Outcomes Using Regression Discontinuity Design. **a**, **h** Scatterplot Analysis: Total Cholesterol and Probability of Adverse Outcomes, Triglycerides and Probability of Adverse Outcomes. **b**, **i** Regression Discontinuity plot: At a triglycerides (TG) level of 1.14 mmol/L, patients exhibited a notable decrease in the risk of adverse outcomes. Similarly, at a total cholesterol (TC) level of 5.34 mmol/L, patients demonstrated a substantial reduction in the risk of adverse outcomes. The dots represent Odds Ratio (OR) values, while the shading represents confidence intervals. **c-e**, **j-i** Sensitivity analysis. **f**, **m** Continuity tests; (**g, n**) Placebo tests: The robustness of the final model results is assessed at various percentile values, including 1, 10, 25, 50, 75, 90 and 99%, respectively

The RDD revealed substantial associations with TC and TG, indicating that patients were at significantly higher risk of adverse outcomes upon reaching cut-off point (all *P* <  0.05, Fig. 3). Sensitivity tests, including Discontinuity estimate and Placebo Tests, were performed, and consistent results were obtained across different bandwidths (Supplementary Table 5, 6). Furthermore, covariates were found to be balanced on both sides of the cut-off point (Supplementary Table 7, 8), supporting the robustness of findings.

### Various fat-related indicators manifest distinct effects over time

A national multicentre prospective cohort study was conducted to validate the association of various fat-related markers with poor prognosis. The study included 1210 healthy individuals and 941 first-time IS patients. The demographic and clinical characteristics of the study population, stratified by BMI, are presented in Table 4. Importantly, patients with different weight statuses exhibited significant clinical outcomes at both 1 and 2 years, indicating that the protective effect of BMI varies over time, being most pronounced during the 1–2-year period. Furthermore, the correlation between obesity metrics in different population groups is depicted in Supplementary Fig. 3 and Supplementary Table 9.

**Table 4:** Baseline characteristics of individuals in the prospective cohort study

Characteristics	Controls (*n* = 1210)	Ischemic Stroke (*n* = 941, BMI, kg/m^2^)			*P*-value
		< 18.5 (*n* = 36)	18.5–24 (*n* = 403)	≥24 (*n* = 502)	
Patient status					
Age, years	61 (54, 65)	66.5 (59, 70.25)	63 (57, 68)	62 (55, 68)	<0.001* 0.023^+^
Sex male, n (%)	650 (53.7%)	25 (69.4%)	247 (61.3%)	306 (61%)	<0.001* 0.598^+^
Smoker, n (%)	284 (23.5%)	14 (38.9%)	122 (30.3%)	163 (32.5%)	<0.001* 0.504^+^
SBP, mmHg	130 (120, 140)	140 (121.5, 160)	140 (126.5, 160)	148 (134, 163.5)	<0.001*< 0.001^+^
DBP, mmHg	80 (74, 86)	80 (76.5, 90)	80 (78, 90)	90 (80, 100)	<0.001*<0.001^+^
Hypertension, n (%)	308 (25.5%)	22 (61.1%)	223 (55.3%)	323 (64.3%)	<0.001* 0.022^+^
Diabetes, n (%)	50 (4.1%)	3 (8.3%)	45 (11.2%)	54 (10.8%)	< 0.001* 0.868^+^
CHD, n (%)	133 (11%)	4 (11.1%)	86 (21.3%)	113 (22.5%)	< 0.001* 0.272^+^
Fat indicators					
TC, mmol/L	5.06 (4.40, 5.70)	4.95 (4.17, 5.51)	4.80 (4.15, 5.45)	5.10 (4.40, 5.80)	0.122* 0.004^+^
TG, mmol/L	1.40 (0.95, 1.90)	1.30 (0.95, 2.10)	1.50 (1.10, 2.10)	1.80 (1.30, 2.40)	< 0.001* < 0.001^+^
HDL, mmol/L	1.24 (1.04, 1.46)	1.30 (1.03, 1.45)	1.18 (1.00, 1.38)	1.11 (0.96, 1.30)	< 0.001* 0.002^+^
BFP, %	31.0 (26.0, 36.9)	21.1 (19.6, 26.0)	26.8 (23.5, 33.8)	32.9 (28.8, 39.6)	0.525* < 0.001^+^
Clinical outcomes					
Six months, n (%)	–	1 (2.78%)	9 (2.23%)	8 (1.59%)	0.727^+^
One-year, n (%)	–	5 (13.9%)	20 (5%)	18 (3.6%)	0.015^+^
Two-year, n (%)	–	8 (22.2%)	45 (11.2%)	40 (8%)	0.011^+^
Three-year, n (%)	–	11 (30.6%)	82 (20.3%)	99 (19.7%)	0.297^+^
Four-year, n (%)	–	14 (38.9%)	112 (27.8%)	147 (29.3%)	0.365^+^
Five-year, n (%)	–	17 (47.2%)	122 (30.3%)	157 (31.3%)	0.110^+^
RFS, week	–	46.2 (33.6, 63)	63 (44.4, 63)	63 (42, 63)	0.080^+^

*: Comparisons between controls and ischemic stroke group. +: Within ischemic stroke subgroup comparisons. The demographic and clinical data were presented as column percentages (%)

**Fig. 4:**
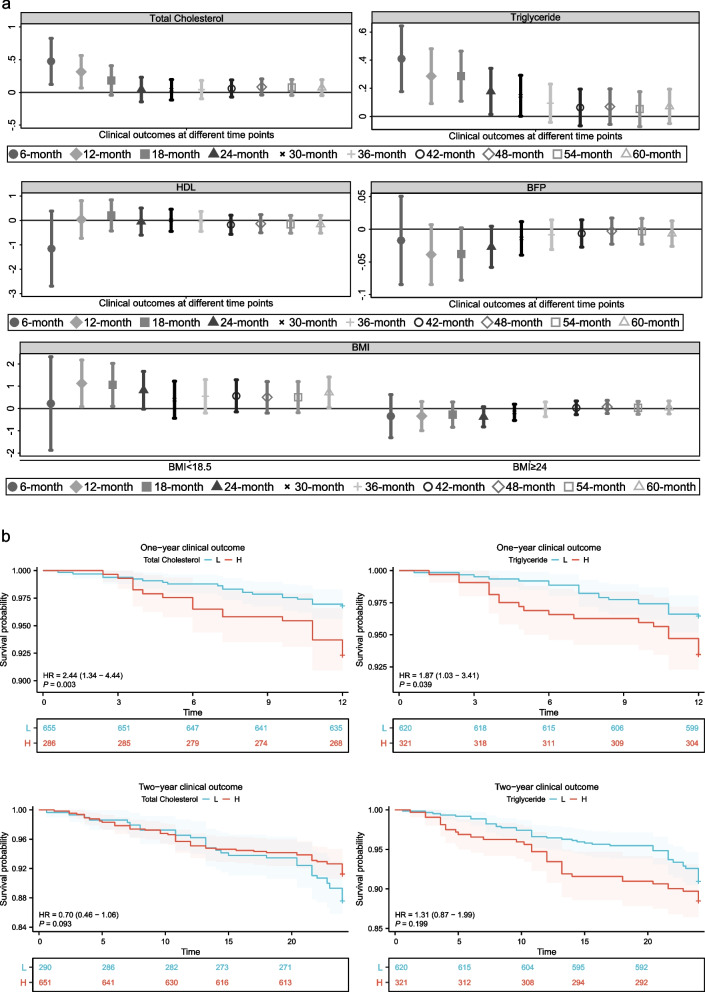
The correlation between various fat-related indicators and clinical outcomes at different time intervals. **a** Exploring the relationship between different fat-related indicators and clinical outcomes at different time points. **b** KM curve of 1-year and 2-year adverse in IS

The investigation involved assessing the association between various obesity indicators and clinical outcomes at different time intervals using GAM. Clinical outcomes were treated as a binomial variable, with the logit function used as the link (Fig. 4a). The findings indicate that TC and TG, both of which are indicators related to obesity, act as risk factors for adverse outcomes in individuals with IS within a one-year timeframe. Furthermore, the impact of both indicators on adverse outcomes diminishes gradually with the extension of the time frame. Additionally, obesity as defined by BMI did not exhibit significant protective effects compared to normal weight (all *P* > 0.05), while wasting emerged as a risk factor over a one to two-year time horizon (12-month: OR = 2.900, 95% CI: (1.108–7.592), *P* = 0.030; 18-month: OR = 3.089, 95% CI: (1.085–8.792), *P* = 0.035). Table 5 presents the model after adjustment for confounding variables, indicating that both TG and TC persist as risk factors 1 year following disease onset. Furthermore, the impact of total cholesterol endured for a longer duration compared to that of triglycerides. Finally, the Kaplan-Meier curve demonstrated that individuals with elevated levels of TC or TG experienced a worse prognosis within a one-year timeframe (Fig. 4b, Supplementary Fig. 4–5).

**Table 5:** Logistic regression analysis of TG and TC linked to unfavorable outcomes

Characteristics	Crude model		Model1		Model2	
	OR (95%CI)	*P*-value	OR (95%CI)	*P*-value	OR (95%CI)	*P*-value
TG						
One-year, n (%)	1.332 (1.097–1.618)	0.004	1.333 (1.098–1.618)	0.004	1.333 (1.098–1.619)	0.004
Two-year, n (%)	1.195 (1.015–1.407)	0.032	1.192 (1.010–1.407)	0.038	1.195 (1.015–1.408)	0.033
Three-year, n (%)	1.098 (0.958–1.258)	0.178	1.190 (0.949–1.253)	0.222	1.191 (0.949–1.254)	0.222
TC						
One-year, n (%)	1.369 (1.069–1.754)	0.013	1.376 (1.074–1.763)	0.012	1.378 (1.074–1.767)	0.012
Two-year, n (%)	1.045 (0.867–1.259)	0.645	1.047 (0.869–1.262)	0.629	1.047 (0.869–1.263)	0.629
Three-year, n (%)	1.042 (0.908–1.197)	0.558	1.046 (0.911–1.202)	0.525	1.047 (0.911–1.202)	0.521

A generalized linear model (GLM) was utilized to perform univariate and multivariate logistic regression, employing a binomial family and logit link. Model1: adjusted for sex and age. Model2: adjusted for sex, age, and hypertension

## Discussion

This study suggests that dyslipidaemia (TG, TC) rather than obesity (BMI, BFP) acts as a significant factor contributing to the unfavourable prognosis of IS patients. Data from the derivation cohort, incorporating a comprehensive range of adiposity measures such as BMI, BFP, HDL, TG, and TC, revealed that TG and TC consistently emerged as predictors of negative clinical outcomes. Failing to support the obesity paradox, the study did not identify a significant protective effect associated with higher BMI values. These conclusions were further reinforced by utilizing robust techniques such as multivariate analysis, PSM and RDD. The results of a rigorous national prospective cohort study underscored that the prognostic impact of dyslipidaemia distinctly presents within the initial year following IS onset and weakens considerably beyond this period, indicating that TG and TC, as markers of dyslipidaemia, continue to represent risk factors for IS.

The novelty of the study lies in its examination of the interplay between lipids, obesity, and prognosis in ischemic stroke patients. Traditionally, obesity has been viewed as potentially impacting the prognosis of stroke patients, with conflicting perspectives on whether it acts as a risk factor for adverse outcomes or as a protective factor [34]. The conventional definition of obesity based on BMI was deemed flawed due to its inability to differentiate between muscle and fat mass, potentially misclassifying individuals. It is crucial to evaluate the distinct components of obesity to accurately assess its impact [12]. While international obesity criteria rely on BMI, this measurement fails to differentiate between fat and muscle proportions in body weight and does not elucidate lipid levels. Consequently, the analysis delved into the effects of distinct obesity components – BMI, BFP, and blood lipids – on the prognosis of IS patients.

Initially, this study identified elevated levels of TG and TC as significant risk factors influencing the prognosis of patients with IS. This conclusion aligns with existing research [13], stressing the correlation between dyslipidaemia and the heightened risk of IS relapse. Hyperlipidemia arises from excessive consumption of a high-cholesterol diet, leading to elevated blood lipid levels [35]. Lipid levels play a role in the development of carotid and intracranial atherosclerosis, additional risk factors for IS. TG and TC are significant lipid components present in the bloodstream. Elevated TG levels may contribute to atherosclerosis development through mechanisms such as excess free fatty acid provision, production of pro-inflammatory cytokines, fibrinogen, coagulation factors, and impaired fibrinolysis [36]. Cholesterol is crucial for cell membranes, brain and nerve cells, bile function, and aids in fat and fat-soluble vitamin absorption. Elevated total cholesterol levels also heighten the risk of atherosclerosis. This finding suggests that monitoring TG and TC levels during the acute and subacute phases of IS crucial, as the highest risk of adverse outcomes occurs within the first year of disease onset. The temporal effect partially accounts for the evolving impact of blood lipid on the prognosis of critically ill patients over time [37, 38].

Furthermore, the current study did not find a significant prognostic effect of obesity, either as a risk factor or a protective factor, in the initial occurrence of IS among the East Asian population. This discrepancy contradicts some previous studies that hint at a protective impact attributed to high BMI in the case of stroke patients [7, 24]. The variation possibly stems from the complex BMI-stroke outcome relationship [6], exacerbated by age and sex differences, implying that generic metrics like BMI are inadequate to illustrate nuanced risk profiles spanning different demographic groups [12, 39]. To delve deeper into this complexity, the study incorporated BFP, an advanced measure that considers BMI, age, and gender. The results confirmed that neither BFP nor BMI exhibited a significant correlation with adverse outcomes in IS cases, raising doubts about obesity’s presumed protective effects.35.

### Study strengths and limitations

The strength of the study is employing a comprehensive and rigorous methodological framework, including retrospective and prospective cohort analyses bolstered by advanced statistical methods (such as PSM and RDD), enhanced the robustness of the study and potentially its applicability to a broader patient population. Additionally, the nationwide multicentre nature of the study allowed for a large and diverse patient population to be included, increasing the generalizability of the findings. By utilizing both retrospective and prospective data, the study provides a comprehensive and reliable assessment of the relationship between fat-related indicators and adverse outcomes in IS patients. A novel approach was employed, integrating a machine learning predictive model using SHAPE values to decipher the impactful contributions of various adiposity measures on IS outcomes. The results confirmed that dyslipidaemia metrics are primary predictors for adverse clinical events. The analysis revealed that body fat percentage, TG, and TC score emerged as the top three contributors to adverse clinical outcomes. These findings provide further evidence supporting the notion that fat-related indicators serve as significant risk factors for adverse outcomes in stroke patients. These methodological strengths contribute to the significance and reliability of the findings, providing valuable insights for clinical practice and future research in this area. The study recognizes its notable strengths and acknowledges inherent limitations, including potential selection bias and confounding variables typical of observational studies. Additionally, while associations between fat-related indices and IS outcomes were established, the study did not delve into the underlying biological mechanisms that could possibly explain these observations. Future research should seek to unveil the biological pathways through which lipid levels and other fat-related factors impact IS patient prognosis, thereby gaining a deeper comprehension of the underpinning pathophysiology and potential intervention targets.

## Conclusions

In conclusion, this study emphasizes the importance of prioritizing lipid management over weight management in individuals with ischemic stroke, highlighting the critical role of dyslipidaemia, particularly elevated TG and TC levels, as significant risk factors for adverse outcomes in IS patients.

### Supplementary Information

**Supplementary material 1.**

**Supplementary material 2.**

**Supplementary material 3.**

**Supplementary material 4.**

**Supplementary material 5.**

**Supplementary material 6.**

## Data Availability

The data and code used in this study could be obtained from the corresponding author upon reasonable request.

## Abbreviations

ANOVA: one-way analysis of variance
BFP: Body fat percentage
BMI: Body Mass Index
CHD: Chronic cardiac disease
CKD: Chronic Kidney Disease
CI: Confidence interval
CRD: Chronic respiratory disease
DBP: Diastolic blood pressure
DTC: DecisionTreeClassifier
GBC: GradientBoostingClassifier
GAM: Generalized additive model
GNB: GaussianNB
HDL: High-Density Lipoprotein
IS: Ischemic stroke
KM: Kaplan-Meier
LR: LogisticRegression
LOWESS: Local weighted regression scatterplot smoothing
PSM: Propensity score matching
RFS: Recurrence-free survival
RF: RandomForestClassifier
RCS: Restricted cubic spline regression
RDD: Regression discontinuity designs
ROC: Receiver operating characteristic
SBP: Systolic blood pressure
SHAP: SHapley Additive exPlanations
TC: Total Cholesterol
TG: Triglyceride
