# Supplementary material for: Dyslipidemia versus obesity as predictors of ischemic stroke prognosis: a multi-center study in China
Source: Lipids Health Dis. 2024 Mar 9;23:72. doi: 10.1186/s12944-024-02061-9 (PMC10924996; doi:10.1186/s12944-024-02061-9)
Supplement: Supplementary file 1 — Supplementary material 1. [file 12944_2024_2061_MOESM1_ESM.pdf]

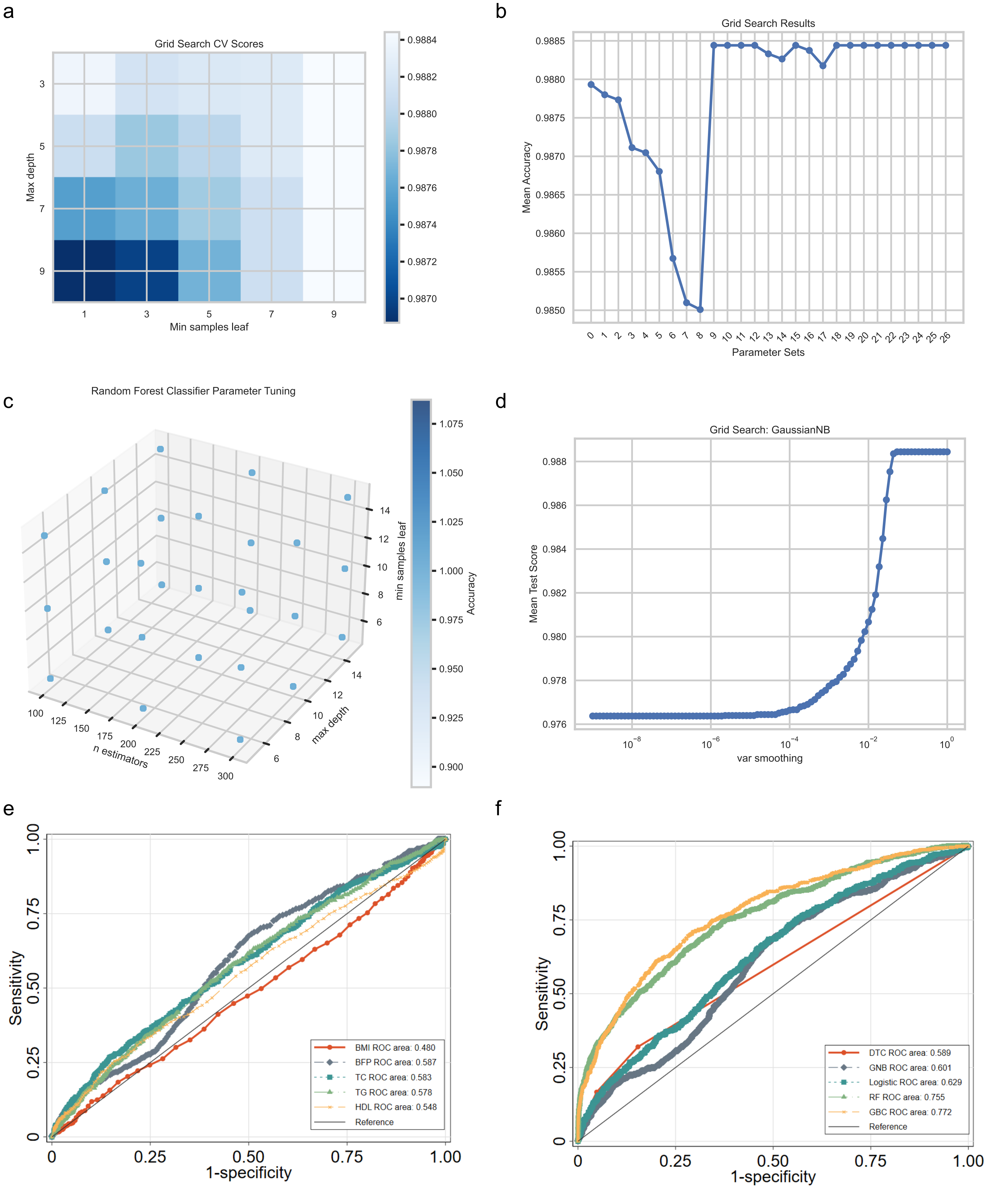

Supplementary figure 1: Tuning process for different machine learning models. (a) Decision Tree Classifier model; (b) Gradient Boosting Classifier model; (c) Random Forest Classifier medel; (d) GaussianNB model; (e) ROC Curves of Different Obesity Indicators Individually Predicting RIS; (f) Roc curves for RIS prediction by different machine learning models
