# Supplementary material for: Dyslipidemia versus obesity as predictors of ischemic stroke prognosis: a multi-center study in China
Source: Lipids Health Dis. 2024 Mar 9;23:72. doi: 10.1186/s12944-024-02061-9 (PMC10924996; doi:10.1186/s12944-024-02061-9)
Supplement: Supplementary file 3 — Supplementary material 3. [file 12944_2024_2061_MOESM3_ESM.pdf]

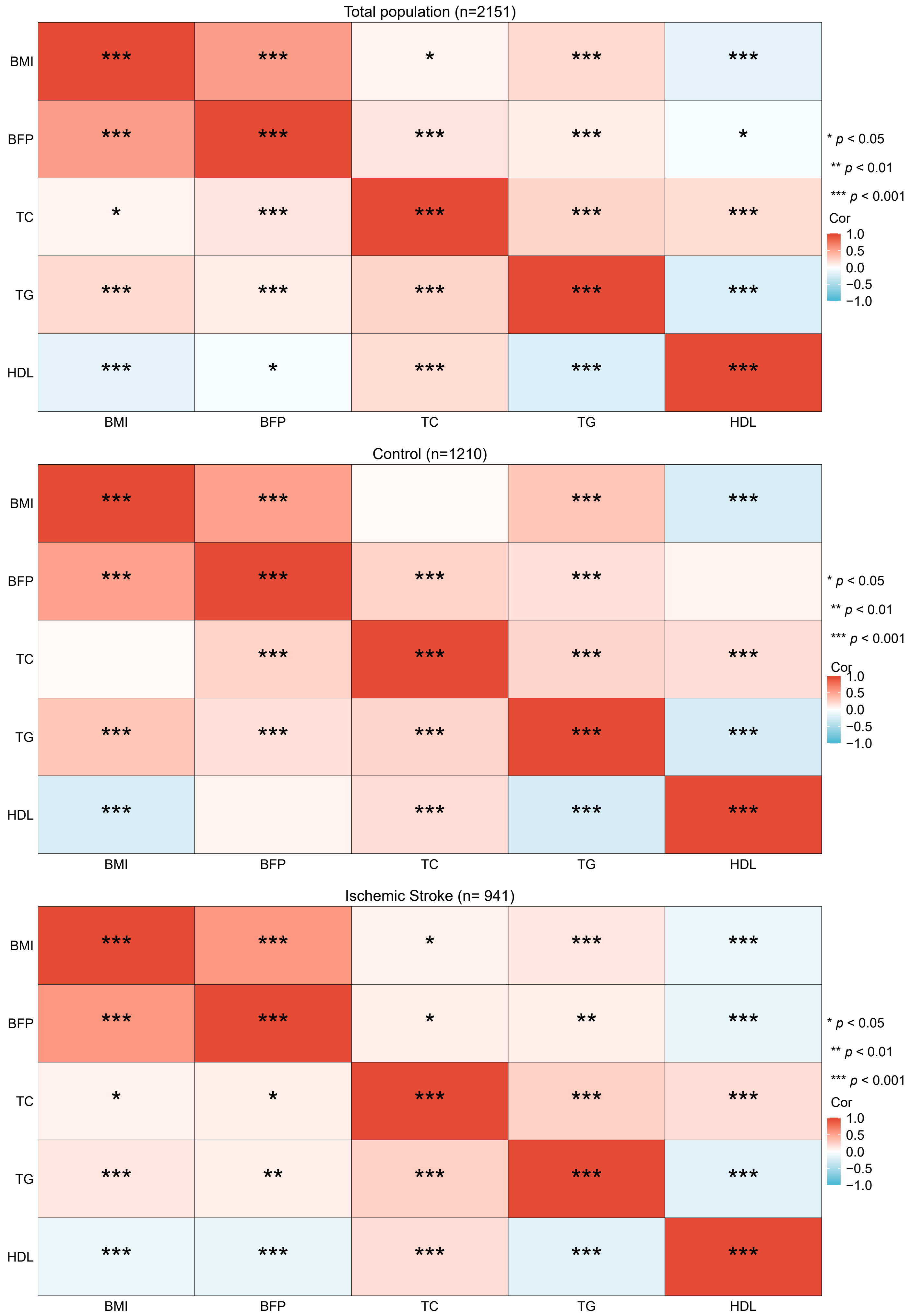

Supplementary figure 3: Heatmap based on spearman correlation coefficients between different clinical data.
