# Supplementary material for: Dyslipidemia versus obesity as predictors of ischemic stroke prognosis: a multi-center study in China
Source: Lipids Health Dis. 2024 Mar 9;23:72. doi: 10.1186/s12944-024-02061-9 (PMC10924996; doi:10.1186/s12944-024-02061-9)
Supplement: Supplementary file 4 — Supplementary material 4. [file 12944_2024_2061_MOESM4_ESM.pdf]

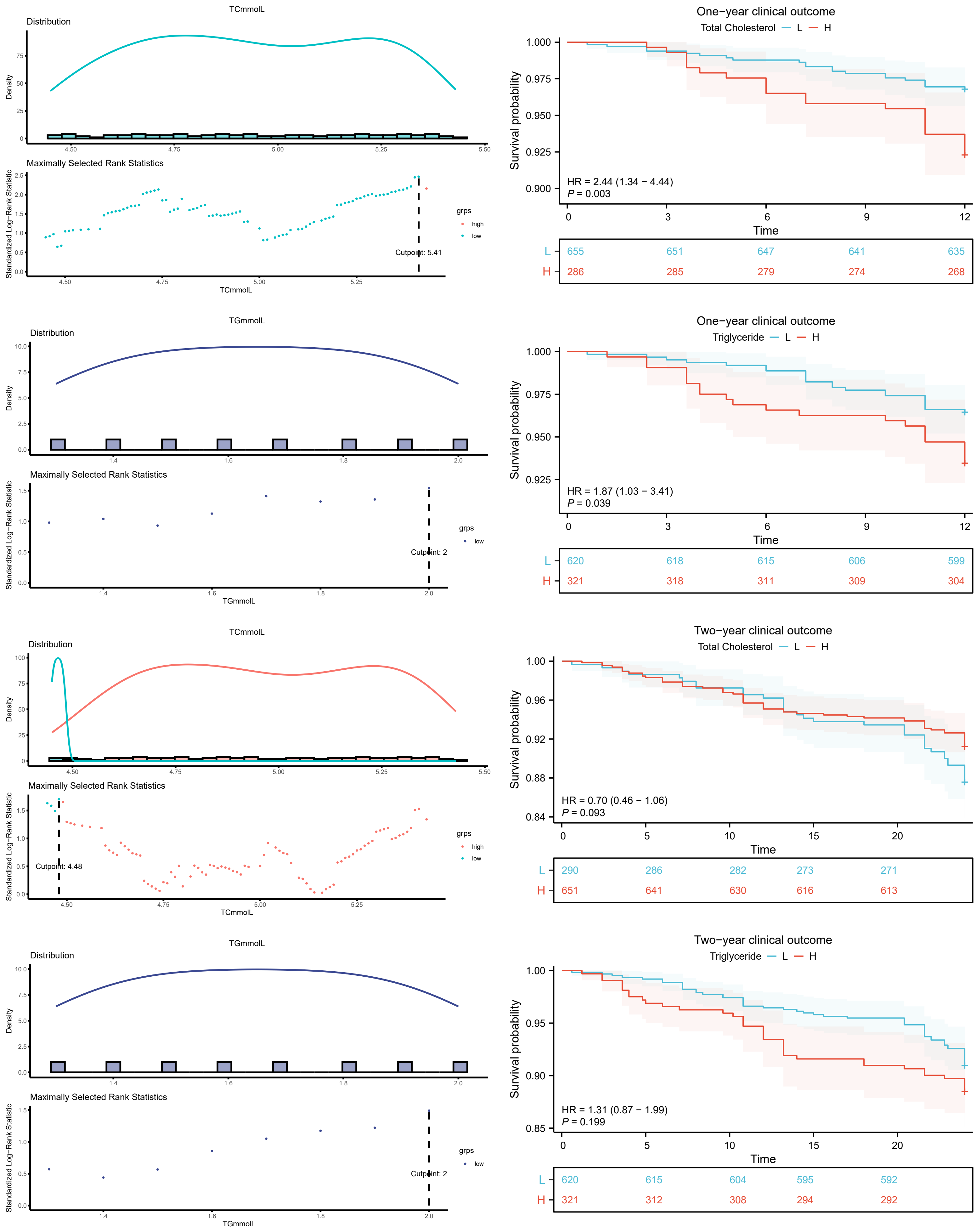

Supplementary figure 4: KM survival curves of the Ischemic Stroke patients. Utilize the 'maxstat' method to identify the optimal cutpoint for each variable. Subsequently, partition the variable values according to the cutpoint obtained from the optimal cutpoint calculation.The "minprop" parameter of the "surv_cutpoint" function (indicating the minimal proportion of observations per group) was established at 30% to mitigate the risk of insufficient patient representation within a specific group.
