# Supplementary figures and images for: Dyslipidemia versus obesity as predictors of ischemic stroke prognosis: a multi-center study in China
Source: Lipids Health Dis. 2024 Mar 9;23:72. doi: 10.1186/s12944-024-02061-9 (PMC10924996; doi:10.1186/s12944-024-02061-9)

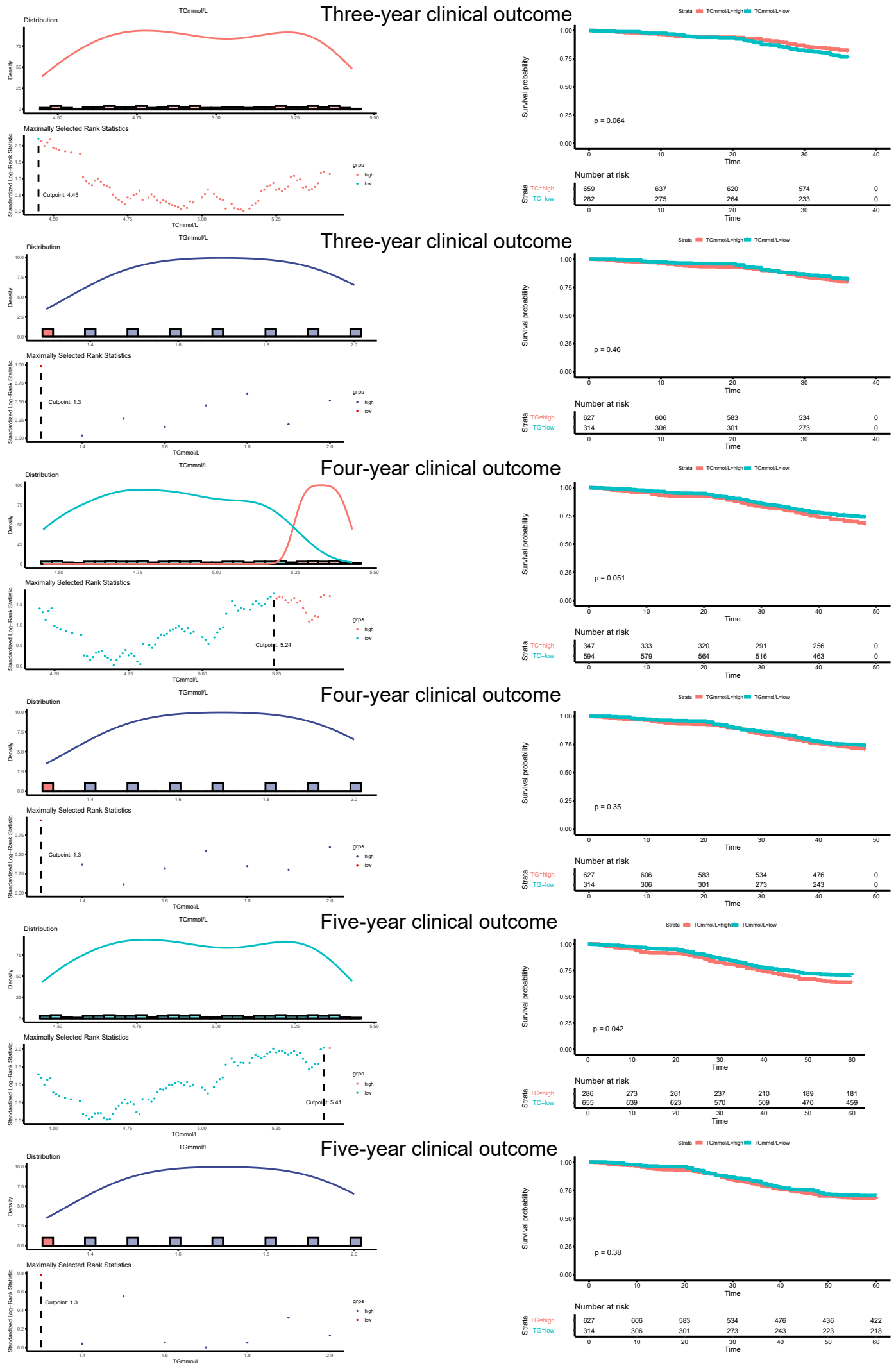

Supplementary figure 5: KM survival curves of the Ischemic Stroke patients.

Supplement: Supplementary file 5 — Supplementary material 5. [file 12944_2024_2061_MOESM5_ESM.pdf]
