# Supplementary material for: Dyslipidemia versus obesity as predictors of ischemic stroke prognosis: a multi-center study in China
Source: Lipids Health Dis. 2024 Mar 9;23:72. doi: 10.1186/s12944-024-02061-9 (PMC10924996; doi:10.1186/s12944-024-02061-9)
Supplement: Supplementary file 6 — Supplementary material 6. [file 12944_2024_2061_MOESM6_ESM.docx]

Supplementary table 1: Baseline characteristics of individuals in the prospective cohort study.

| Characteristics | Controls (n=1210) | Ischemic Stroke (n= 941) | P-value |
| --- | --- | --- | --- |
| Patient status |  |  |  |
| Age, years | 61 (54, 65) | 63 (56, 68) | < 0.001 |
| Sex male, n (%) | 650 (53.7%) | 578 (61.4%) | < 0.001 |
| Smoker, n (%) | 284 (23.5%) | 299 (31.8%) | < 0.001 |
| SBP, mmHg | 130 (120, 140) | 140 (130, 160) | < 0.001 |
| DBP, mmHg | 80 (74, 86) | 85 (80, 95) | < 0.001 |
| Comorbidities |  |  |  |
| Hypertension, n (%) | 308 (25.5%) | 568 (60.4%) | < 0.001 |
| Diabetes, n (%) | 50 (4.1%) | 102 (10.8%) | < 0.001 |
| CHD, n (%) | 133 (11%) | 203 (21.6%) | < 0.001 |
| Fat-related indicators |  |  |  |
| TC, mmol/L | 5.06 (4.4, 5.7) | 4.9 (4.3, 5.7) | 0.122 |
| TG, mmol/L | 1.4 (0.9525, 1.9) | 1.7 (1.2, 2.3) | < 0.001 |
| HDL, mmol/L | 1.24 (1.04, 1.46) | 1.14 (0.98, 1.34) | < 0.001 |
| BMI, kg/m^2^ | 24.0 (22.0, 26.0) | 24.2 (21.9, 26.4) | 0.205 |
| BFP, % | 31.0 (26.0, 36.9) | 30.4 (25.9, 36.8) | 0.579 |

Note. The demographic and clinical data were presented as column percentages (%). Abbreviations: BFP: Body fat percentage; BMI: Body Mass Index; CHD: Chronic cardiac disease; DBP, diastolic blood pressure; HDL: High-Density Lipoprotein; RIS: Recurrent ischemic stroke; SBP, Systolic blood pressure; TC: Total Cholesterol; TG: Triglyceride.

Supplementary table 2: Predictive performance of different Fat indicators in the derivation cohort.

| Characteristics | ROC | 95% CI | P-value | Bonferroni |
| --- | --- | --- | --- | --- |
| BMI | 0.480 | 0.454 - 0.506 | Ref. | Ref. |
| BFP | 0.587 | 0.564 - 0.611 | 0.000 | 0.000 |
| TC | 0.583 | 0.558 - 0.609 | 0.000 | 0.000 |
| TG | 0.578 | 0.553 - 0.602 | 0.000 | 0.000 |
| HDL | 0.548 | 0.521 - 0.574 | 0.002 | 0.009 |

Note: The areas under curves (AUC) of ROC were compared using Delong's test, and multiple comparisons were corrected using the Bonferroni correction. Abbreviations: BFP: Body fat percentage; BMI: Body Mass Index; HDL: High-Density Lipoprotein; TC: Total Cholesterol; TG: Triglyceride.

Supplementary table 3: Comparison of predictive performance of different machine learning models.

| Models | ROC | 95% CI | P-value | Bonferroni |
| --- | --- | --- | --- | --- |
| DTC | 0.589 | 0.568 - 0.610 | Ref. | Ref. |
| GNB | 0.601 | 0.578 - 0.623 | 0.419 | 1.000 |
| Logistic | 0.629 | 0.606 - 0.653 | 0.002 | 0.007 |
| RF | 0.755 | 0.733 - 0.777 | 0.000 | 0.000 |
| GBC | 0.772 | 0.751 - 0.793 | 0.000 | 0.000 |

Note: The areas under the ROC curves (AUCs) were compared using Delong's test, and multiple comparisons were corrected using the Bonferroni correction. Abbreviations: DTC, Decision Tree Classifier; GBC, Gradient Boosting Classifier; GNB, Gaussian Naive Bayes; Logistic, Logistic Regression; RF: Random Forest.

Supplementary table 4: Collinearity diagnostics of variables for multivariate analysis.

| Variable | VIF | SQRT-VIF | Tolerance | R-Squared |
| --- | --- | --- | --- | --- |
| Age | 1.190 | 1.090 | 0.840 | 0.160 |
| SBP | 2.150 | 1.460 | 0.466 | 0.534 |
| DBP | 2.040 | 1.430 | 0.490 | 0.510 |
| TC | 1.180 | 1.090 | 0.846 | 0.154 |
| TG | 1.210 | 1.100 | 0.828 | 0.172 |
| Diabetes | 1.240 | 1.110 | 0.806 | 0.194 |
| CHD | 1.110 | 1.050 | 0.900 | 0.100 |
| CKD | 1.080 | 1.040 | 0.926 | 0.074 |
| CRD | 1.100 | 1.050 | 0.909 | 0.091 |
| Insulin use | 1.240 | 1.110 | 0.805 | 0.196 |

Note: A Variance Inflation Factor (VIF) greater than 10 was considered as the threshold for indicating significant collinearity. Abbreviations: TG: Triglyceride; CHD: Chronic cardiac disease; CRD: Chronic respiratory disease.

Supplementary table 5: Robustness checks for triglyceride levels in a regression discontinuity design.

| Triglyceride | Coefficient | Standard errors | z | P-value | 95% CI |
| --- | --- | --- | --- | --- | --- |
| Lwald | 0.046 | 0.018 | 2.560 | 0.010 | 0.011 -0.082 |
| Lwald*0.5 | 0.052 | 0.024 | 2.140 | 0.032 | 0.004 -0.100 |
| Lwald*2 | 0.033 | 0.015 | 2.200 | 0.028 | 0.004 -0.063 |

Note: The study examines the sensitivity of the results to bandwidth choice in a Regression Discontinuity Design. Three different bandwidth values are selected: the Optimal Bandwidth, 2 times the Optimal Bandwidth, and half of the Optimal Bandwidth. This sensitivity analysis aims to assess the robustness of the estimated treatment effects under different choices of bandwidth. Abbreviations: Lwald: Limdep Wald.

Supplementary table 6: Robustness checks for total cholesterol levels in a regression discontinuity design.

| Total Cholesterol | Coefficient | Standard errors | z | P-value | 95% CI |
| --- | --- | --- | --- | --- | --- |
| Lwald | 0.073 | 0.031 | 2.360 | 0.018 | 0.012 - 0.134 |
| Lwald*0.5 | 0.100 | 0.038 | 2.640 | 0.008 | 0.026 - 0.174 |
| Lwald*2 | 0.051 | 0.024 | 2.120 | 0.034 | 0.004 - 0.098 |

Note: The study examines the sensitivity of the results to bandwidth choice in a Regression Discontinuity Design. Three different bandwidth values are selected: the Optimal Bandwidth, 2 times the Optimal Bandwidth, and half of the Optimal Bandwidth. This sensitivity analysis aims to assess the robustness of the estimated treatment effects under different choices of bandwidth. Abbreviations: Lwald: Limdep Wald.

Supplementary table 7: Balance Checks for triglyceride levels in a regression discontinuity design. (Triglyceride cutoff point= 1.14 mmol/L)

| Triglyceride | Coefficient | Standard errors | z | P-value | 95%CI |
| --- | --- | --- | --- | --- | --- |
| BMI | 0.008 | 0.325 | 0.020 | 0.981 | -0.629 - 0.644 |
| BFP | 1.482 | 0.977 | 1.520 | 0.129 | -0.432 - 3.396 |
| TC | -0.017 | 0.173 | -0.100 | 0.922 | -0.357 - 0.323 |
| HDL | -0.087 | 0.062 | -1.420 | 0.157 | -0.208 - 0.034 |

Supplementary table 8: Balance Checks for Total Cholesterol levels in a regression discontinuity design. (TC cutoff point= 5.34 mmol/L)

| Total Cholesterol | Coefficient | Standard errors | z | P-value | 95%CI |
| --- | --- | --- | --- | --- | --- |
| BMI | -0.629 | 0.407 | -1.540 | 0.122 | -1.427 - 0.169 |
| BFP | 1.420 | 1.013 | 1.400 | 0.161 | -0.565 - 3.405 |
| TG | -0.416 | 0.285 | -1.460 | 0.144 | -0.974 - 0.142 |
| HDL | 0.027 | 0.067 | 0.400 | 0.690 | -0.105 - 0.159 |

Supplementary table 9: Partial and semi partial correlations of BMI with Variable

| Variable | Partial corr. | Semi partial corr. | Partial corr.^2 | Semi partial corr.^2 | P-value |
| --- | --- | --- | --- | --- | --- |
| BFP | 0.566 | 0.556 | 0.321 | 0.309 | 0.000 |
| TG | 0.089 | 0.072 | 0.008 | 0.005 | 0.001 |
| TC | -0.029 | -0.023 | 0.001 | 0.001 | 0.277 |
| HDL | -0.134 | -0.110 | 0.018 | 0.012 | 0.000 |

Abbreviations: Partial corr., Partial correlation coefficient; Semi partial corr., Semi partial correlation coefficient; Partial corr.^2, Squared partial correlation coefficient; Semi partial corr.^2, Squared semi partial correlation coefficient.
